# Soy Isoflavones Prevent Bone Quality Loss Induced by High‐Fat Diet in Rats Through Epigenetic Modifications

**DOI:** 10.1096/fj.202500767RRR

**Published:** 2025-10-22

**Authors:** Perry C. Caviness, Beau Belcher, Oxana P. Lazarenko, Jennifer F. Chen, Michael L. Blackburn, Jin‐Ran Chen

**Affiliations:** ^1^ Arkansas Children's Nutrition Center Little Rock Arkansas USA; ^2^ Department of Pediatrics University of Arkansas for Medical Sciences Little Rock Arkansas USA; ^3^ Undergraduate Pre‐Medical Program, University of Arkansas at Fayetteville Fayetteville Arkansas USA

**Keywords:** high fat diet, obesity, osteoclast, soy isoflavones

## Abstract

Chronic consumption of a Western‐style diet, high in saturated fats and cholesterol, disrupts energy metabolism, leading to obesity and insulin resistance. In contrast, isoflavone (Isof)‐rich soy has been shown to benefit connective tissue metabolism. However, the potential of soy Isof dietary supplementation to counteract the effects of a high‐fat diet (HFD) on bone development, morphology, and remodeling remains poorly understood. In this study, 24‐day‐old male Sprague–Dawley rats were fed a HFD containing 45% fat and 0.25% cholesterol for 8 weeks, leading to significant increases in body weight, long bone length, bone marrow adiposity, and insulin resistance when compared to control diet (AIN‐93G) rats. Using micro‐CT and three‐point bending tests, we found that supplementation of HFD with dietary soy Isof (NovasoyR 400: 352 mg Isof/g; Genistein, Daidzein, Glycitein ratio of 1.3:1:0.15 identical to that found in soybeans) prevented HFD‐induced reductions in bone mass, including bone volume, trabecular number, density, and strength. These bone‐preserving effects were associated with decreased non‐esterified free fatty acid (NEFA) levels and increased alkaline phosphatase activity in serum and bone marrow plasma. Isof supplementation also ameliorated HFD‐induced increases in the expression of Ezh2 and H3k27me3 levels (gene silencing epigenetic mark catalyzed by Ezh2) in bone and blocked the elevated expression of osteoclastic marker NFATc1 in bone marrow cells. A significant inverse correlation between bone mineral density (BMD) and DNA methylation marker 5‐methylcytosine (%5‐mC), as well as a positive correlation between serum NEFA levels and Ezh2 expression, were observed. Furthermore, Isof supplementation inhibited HFD‐induced increases in bone DNA methylation. These results suggest that: (1) HFD disrupts bone and adipose tissue development and remodeling in post‐weanling juvenile rat models; (2) Isofs may protect against high‐fat diet‐induced bone impairments. We hypothesize that the molecular mechanisms underlying Isof's bone protective effects may involve modulation of histone and DNA methylation through regulation of Ezh2 levels.

## Graphical abstract

A diet high in fat and cholesterol, especially when consumed early in life, can lead to negative outcomes such as obesity and impaired bone development. This can increase the risk of fractures and bone loss later in life. In young rats, adding soy isoflavones (Isof) ‐ a type of compound found in soy foods that resemble estrogen ‐ into a high‐fat diet helped prevent these harmful effects on bone health. We hypothesize that the beneficial effects of Isofs on bone may be linked to chemical changes in DNA that regulate how genes are expressed known as DNA methylation.

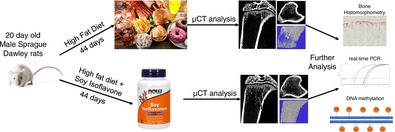

**Abbreviations**
Akt: serine/threonine‐specific protein kinase
ALP: alkaline phophatase
GAPDH: glyceraldehyde 3‐phosphate dehydrogenase
HFD: high fat diet
IGF1: insulin‐like growth factor 1
IRS1: Insulin receptor substrate 1
Isof: soy isoflavones
JNK: c‐Jun N‐terminal kinase
NEFA: non‐esterified free fatty acids
OGTT: oral standard glucose tolerance test
PND: postnatal day

## 1 Introduction

Nutrition, sex hormones, local paracrine molecules, and physical activity are all known to significantly influence bone modeling and maturation during early life [1, 2]. Recent evidence highlights the role of dietary metabolites—derived from food components digested by gut microbiota—in regulating bone turnover. Excessive consumption of a “Western” diet—characterized by high levels of saturated fats and cholesterol (high‐fat diet: HFD) during early prepubertal life is strongly linked to the development of obesity [3, 4]. Research has demonstrated that feeding HFD to rodents induces systemic insulin resistance and metabolic syndrome. This can result in increased bone marrow adiposity and decreased bone quality [1].

Early life dietary interventions may be critical to mitigate the negative effects of HFD on bone. In rats, dietary supplementation with soy protein isolate (SPI), the sole protein source in soy infant formula, was shown to alleviate metabolic syndrome via improvement of lipid and cholesterol homeostasis [5]. Our group has found that in rats, SPI dietary supplementation of HFD can ameliorate HFD‐induced negative effects on bone [6]. In addition, in rats, it was shown that either short‐term consumption of SPI early in life or long‐term throughout life can minimize bone loss following ovariectomy (OVX) [7].

The bone protective effects of SPI diet are thought to be due to high concentrations of isoflavones (Isof), molecules structurally similar to 17β‐estradiol (E2) [8, 9]. However, comparison of short‐term SPI dietary supplementation with subcutaneous E2 injection in prepubertal rats revealed differential effects between the two. In addition, combination of SPI diet and E2 injection revealed that SPI diet may have some anti‐estrogen effects [10, 11]. Isof treatment of osteoblastic cell lines increased osteoblast proliferation, reduced insulin resistance, and blocked nonesterified free fatty acid induced increases in senescence pathways [6, 7, 12]. In rat models, Isof dietary consumption was also found to increase bone turnover markers pyridinoline, CTX1, Alp, and P1NP; as well as decrease bone surface area occupied by adipose tissue and increase calcium transport into bone tissue [13, 14, 15]. Femur levels of magnesium, another essential nutritional factor for bone health, were also shown to be increased in rat bone tissue following consumption of AIN 93 M diet supplemented with tempeh, a soybean product rich in Isofs [16]. However, Isof consumption did not affect iron levels when compared with soybean flour [17]. Human studies in both pre‐ and postmenopausal women found Isof consumption can attenuate bone loss [18]. However, the mechanism by which Isof can regulate bone remodeling remains poorly understood.

The bone protective effects of Isofs may be through epigenetic modifications as Isofs have been shown to affect both DNA methylation and histone modification [19, 20, 21]. Our investigations into the relationship between epigenetics and bone have shown a negative relationship between HFD and healthy bone metabolism. Embryotic calvaria cells from the fetus of pregnant mice fed a HFD had increased presence of the repressive epigenetic mark H3K27me3 in the gene body of SATB2, a transcription factor necessary for osteoblast differentiation. Furthermore, it was found that the osteoblast cell specific deletion of Ezh2 (enhancer of zeste homologue 2: a histone lysine methyltransferase which catalyzes the formation of H3K27me3) in mice resulted in increased trabecular bone mass when compared to controls [22]. Follow‐up studies revealed increased H3K27me3 presence in gene bodies (and subsequent decrease in mRNA expression) of osteogenesis promoting genes *Pthlh*, *Bmp6*, and *Col2a1* in both cells and bone tissue. Epigenetic changes were present well into adulthood [23]. In addition to osteoblasts, we also found that the deletion of Ezh2 from pre‐osteoclast myeloid cells in mice led to significant increases in trabecular bone as well as increased expression of osteoclast suppressive genes *Irf8*, *MafB*, and *Arg1* [24].

In this study, we seek to further examine the bone protective effects of Isof by investigating if dietary supplementation of HFD with commercially available Isof (NovasoyR 400: 352 mg Isof/g; genistein: daidzein: glycitein ratio 1.3:1:0.15) can mitigate bone‐destructive effects in a post‐weanling juvenile rat model. While there are studies into Isof supplementation to attenuate sex steroid deficiency‐induced bone loss, Isof effects on alleviating HFD‐induced bone loss are lacking [7, 25, 26]. In addition, we seek to understand how dietary metabolites may regulate Ezh2 expression in bone. These insights are essential to determine whether Ezh2‐induced bone degradation can be prevented by dietary supplementation with Isof or other nutritional factors. For this reason, we examined the effect of Isof dietary supplementation on DNA methylation in bone tissue and hypothesize that the bone protective effects of Isof may involve modulation of histone and DNA methylation through regulation of Ezh2 levels.

## 2 Materials and Methods

### 2.1 Animals and Diets

Male Sprague–Dawley rats were purchased from Harlan Industries (Indianapolis, IN), arrived on postnatal day (PND) 20, and acclimated until PND 24. To assess the effects of soy isoflavone dietary supplementation on ameliorating HFD‐induced negative effects on bone, rats were fed one of four diets: (1) a standard low fat (5%) AIN‐93G diet formulated with casein as the sole protein (Control); (2) a high‐fat/high‐cholesterol containing 18.8 MJ/g energy, 195 g/kg casein protein, 483 g/kg carbohydrate, 210 g/kg anhydrous milk fat, 2.5 g/kg cholesterol, and 50 g/kg cellulose fiber (HFD); (3) Novasoy 400 from ADM Nutrition (40% min isoflavone content; 52% genistein: 218 mg/kg, 43% daidzein: 182.37 mg/kg, and 5% glycitein: 21.78 mg/kg) was mixed into the Control diet from (1) (Control + Isof); (4) Novasoy 400 was mixed into the HFD from (2) (HFD + Isof). Due to having the highest composition of isoflavones present in the SPI diet, to investigate the bone protective effects of isoflavones in HFD rat models, Novasoy 400 dosage (Control + Isof: 19.3 mg/day; HFD + Isof: 21 mg/day) was normalized to give genistein intake equivalent to rats fed SPI supplemented AIN‐93G diet (218 mg/kg/day) or SPI supplemented HFD diet (253 mg/kg/day) [5]. Rats were fed their respective diets from PND 24 to PND 68. Control and HFD rats were pair‐fed to Control + Isof and HFD + Isof rats, respectively (total calories), both of whom had *ad libitum* access to food. All groups had ad libitum access to water. For rats fed HFD (HFD and HFD + Isof), the diet was changed out every 2 days. To ensure pair feeding is matched by age, simple randomization was used within a staggered enrollment to assign *N* = 10 per each dietary group.

Rats were housed in an Association for Assessment and Accreditation of Laboratory Animal Care‐approved animal facility at the Arkansas Children's Hospital Research Institute with constant humidity and lights on from 06:00–18:00 h at 22°C. At PND24, rats were assigned 5 per cage. As rats got older and gained weight, they were separated into other cages (generally 2 per cage, combined mass of rats/cage less than 1 kg) with rats heavier than 500 g housed solo. All animal procedures were approved by the Institutional Animal Care and Use Committee at University of Arkansas for Medical Sciences (UAMS, Little Rock, AR). Rat body weights were monitored once per week. Seven days prior to killing, rats were given an oral standard glucose tolerance test (OGTT) and tail blood was taken as described previously [27]. At the completion of the experiment, rats were anesthetized by injection with 100 mg Nembutal/kg body weight (Avent Laboratories), followed by decapitation and collection of bone tissue (tibia, femur and vertebrae) and serum. Serum insulin concentrations were measured using an ELISA (EZRMI‐13 K) for rat serum (Linco Research). Serum glucose was measured using the glucose oxidase method (IR070, Synermed).

### 2.2 Bone μCT and Three‐Point Bending Analysis

Micro‐computed tomography (CT) measurements of tibia (and L3‐L5 spine vertebrae) from the four different rat diet groups (*N* = 9) were evaluated using a Skyscan microCT scanner (SkyScan 1272). Tibia and vertebrae were cleaned of muscle tissue and stored in formalin for at least 24 h prior to scanning. For μCT, the region of interest (ROI) was selected to include the entire epiphysis and metaphysis (including the growth plate), which contains metabolically active trabecular bone. A high percentage of trabecular bone surface is exposed to bone marrow; as such, if HFD or Isof dietary supplementation were to impact bone cell production leading to an effect on bone quantity/quality, it would be most likely observed in areas with high trabecular bone such as the growth plate [28, 29]. Due to these factors, for rats in this study, it was determined that this region was of high importance for further understanding the anti‐bone deterioration effects of soy isoflavones. Images were obtained at 70 kV X‐ray tube voltage and 142 μA current, from a 0.5 mm aluminum filter. Total exposure time was 690 ms per image, and image pixel size was 10 μm. For each specimen, a series of 4589 projection images were obtained (a rotation step of 0.4°, Frame averaging: on, averaging 10 frames). Images were reconstructed using NRecon software (Skyscan). Random movement and flat field correction were turned on, and beam hardening correction was set to 38%. Total bone volume (BV), tissue volume (TV), bone volume fraction (BV/TV %), bone surface density (BS/TV mm^2^), trabecular thickness (Tb Th, mm), trabecular separation (Tb Sp, mm), trabecular number (Tb N, 1/mm), and bone mineral density (BMD) were calculated using Skyscan provided software and averaged for each treatment group.

Immediately following tissue collection, femurs are frozen until needed for three‐point bending analysis. For three‐point bending analysis, femurs (*N* = 9) were cleaned of muscle tissue. The three‐point bending test was performed on femurs at room temperature using a miniature bending apparatus with the posterior femoral surface lying on lower supports (7 mm apart) and the left support immediately proximal to the distal condyles. Load was applied to the anterior femoral surface by an actuator midway between the two supports moving at a constant rate of 3 mm/min to produce a physiological in vivo strain rate of 1% for the average murine femur. Maximum load (N) and displacement (mm) were recorded. The external measurements (length, width, and thickness) of the femur were recorded with a digital caliper. We measured the moment of inertia in the midshaft of the femur using μCT (model μCT40, Scanco Medical). The mechanical properties were normalized for bone size, and ultimate strength and stress (N/mm2; in megapascals and MPa) were calculated.

### 2.3 Bone Histological Analysis

von Kossa and TRAP staining on paraffin embedded tibia sections were carried out using standard protocols from von Kossa Stain kit (ab150687) and leukocyte acid phosphatase kit (TRAPase^+^: Millipore‐Sigma, 386A). MacNeal tetrachrome counterstain was performed for von Kossa stained slides. von Kossa and TRAPase stained tibias were images using an epifluorescent microscope (Nikon Eclipse T/2). For von Kossa staining, ROI at 4X magnification was selected according to similar criteria used for μCT analysis [28, 29]. For TRAPase staining, 10X magnifications of regions in tibia with minimal tissue damage was performed. From the 10X magnification, best representative regions of area under growth plate were selected as ROI for quantification of TRAPase^+^ cells (*N* = 3). TRAPase^+^ cells within this region were normalized to ROI area, approximately 893 450 μm^2^.

### 2.4 Bone Marrow Plasma ALP Activity, L3‐L5 β‐Gal Activity and Serum NEFA Measurements

Bone marrow plasma was prepared at the time of tissue harvest. Bone marrow was flushed out from the femur using 300 μL of PBS, vortexed, and centrifuged (1700 g). Supernatant was collected as bone marrow plasma. Bone formation marker alkaline phosphatase (ALP) activity levels in bone marrow plasma were measured using a colorimetric assay. A 96‐well tissue culture‐treated plate was rinsed with HEPES buffer prior to the addition of 50 μL bone marrow plasma. Following the addition of bone marrow plasma, 75 μL of alkaline buffer solution (Sigma) equilibrated to room temperature was added to the wells. Immediately before loading the tissue culture plate into the plate reader, 75 μL of a reconstituted phosphatase substrate (Sigma, 4‐Nitrophenyl phosphate disodium salt hexahydrate) was added to each well. Following the addition of the substrate, absorbance at 410 nm was observed at 0 and 4 min using a Polarstar Omega plate reader. ALP activity was calculated and normalized to the total amount of protein loaded per well (*N* = 9). Senescence‐associated β‐galactosidase (β‐Gal) activity from L3‐L5 protein lysate was performed using a β‐galactosidase enzyme assay kit (Promega) according to the manufacturer's instructions (*N* = 9). Levels of non‐esterified fatty acids (NEFA) present in rat serum were measured using Wako Diagnostics (Wako Chemicals USA Inc., VA, USA) in vitro enzymatic colorimetric assay for detecting NEFA present in serum (*N* = 9).

### 2.5 Western Blotting

Bone tissue proteins were extracted from L3 to L5 vertebrae for western immunoblot analysis using cell lysis buffer as previously described [30]. Briefly, after cleaning surrounding connective tissues, vertebrae were homogenized in 500 μL RIPA buffer using metal beads and Precellys 24 homogenizer (6000 rpm, 40 s). Following homogenization, tissue was kept on ice for 45 min (vortex every 10 min), centrifuged (14000*g*, 15 min, 4°C) with supernatant collected as protein lysate. Western blot analyses were performed using standard protocols (*N* = 5). The following antibodies were used: H3k27me3 (#07‐449, Millipore), Ezh2 (#5346, Cell Signaling). Secondary antibodies were purchased from Santa Cruz Biotechnology (Goat anti‐rabbit IgG sc‐2004, Goat anti‐mouse IgG sc‐2005). Amido black staining (Sigma‐Aldrich) was used for loading control. Blots were developed using chemiluminescence (PIERCE Biotechnology) according to the manufacturer's recommendations.

### 2.6 RNA Isolation, Real‐Time Reverse Transcription‐Polymerase Chain Reaction

RNA from L3‐L5 vertebrae and femur bone marrow were isolated and extracted using TRI Reagent (MRC Inc., Cincinnati, OH) according to the manufacturer's recommendation. Briefly, bone (L3‐L5 vertebrae) was cleaned of surrounding tissue, placed in 1 mL of TRI Reagent, and homogenized using metal beads and a Precellys 24 homogenizer (6500 rpm, 20 s, twice). Following homogenization, 100 μL of 1‐bromo‐3‐chloropropane (BCP) is added, and homogenized tissue is centrifuged (14,000 rpm, 15 min, 4°C). Supernatant is collected, mixed with an equal volume of isopropanol, and centrifuged again (14,000 rpm, 15 min, 4°C). Supernatant is removed, and pellet is washed twice (800 μL, 75% ethanol, centrifuge at 14,000 rpm, 3 min, 4°C). Following the TRI Reagent procedure, residual ethanol is removed, pellet is air dried for 5 min, resuspended in 100 μL of DEPC treated H_2_O, and purified using DNase digestion followed by column cleanup (QIAGEN mini columns). For femur bone marrow RNA isolation, the same procedure used for L3‐L5 vertebrae is performed. However, prior to TRI Reagent addition, bone marrow is first flushed from femur using the following procedure. Femur is cut using sterile surgical scissors at the epiphysis at both ends, exposing the bone shaft. Open femur is then placed into an Eppendorf tube with 1 mL of PBS and centrifuged (8000 rpm, 5 min, 4°C). Empty bone is removed, 1 mL of TRI is added, and the procedure is performed as previously described. Reverse transcription was carried out using an iScript cDNA synthesis kit from Bio‐Rad (Hercules, CA). All primers for real‐time PCR analysis used in this report were designed using Primer Express software 2.0.0 (Applied Biosystems) and are listed in Supplemental Table S1 (*N* = 9).

### 2.7 %5‐mC Measurement Assay for DNA Methylation

Genomic DNA from rat L3‐L5 vertebrae was isolated using the Qiagen DNeasy Blood and Tissue Kit (Cat. # 69556). First, tissue was homogenized in 180 μL of buffer ATL and 20 μL of proteinase K using metal beads and a tissue homogenizer (6500 rpm, 20 s, twice). Following this, the manufacturer's protocol was followed. Genomic DNA concentration and purity (A260/A280) were determined using a Polarstar Omega plate reader. The %5‐mC was determined using the MethylFlash Global DNA Methylation (5‐mC) ELISA Kit from EpiGentek. Optical density at 450 nm was found using a Polarstar Omega plate reader. %5‐mC was found for each sample using the standard curve (*N* = 9). The average of all readings within each group was found to give a final %5‐mC for each diet. Correlations between %5‐mC and spine BMD, tibia BMD, L3‐L5 vertebrae Ezh2 mRNA levels, and NEFA serum concentration as well as L3‐L5 vertebrae Ezh2 mRNA levels and NEFA serum concentration were found using the correlation function in GraphPad Prism 9.0 (*N* = 7–9).

### 2.8 Statistical Analyses

Based on our previous analysis when comparing the effects of 5% BB supplementation using a sample size of 8 rats/group, the ES(f) from comparing tibial cortical BMD between blueberry diet‐fed rats and those on normal chow was 0.89, whereas an ES(f) = 1.34 was observed when comparing tibial trabecular BMD between the two groups [31]. Hence, with a sample size of 7–9 rats per group in our current study, we should have sufficient power to detect meaningful differences. Numerical variables were expressed as means ± SD (Standard Deviation) using GraphPad Prism 9.0 (GraphPad Software Inc., San Diego, Ca, USA). Comparisons between groups were performed using two‐way ANOVA and corrected for multiple comparisons by Tukey's post hoc test. Assumptions for two‐way ANOVA were confirmed with corresponding statistical tests in GraphPad Prism 9.0 (variance, independence, normality). The critical *p* value for statistical significance was *p* = 0.05.

## 3 Results

### 3.1 Isoflavones Ameliorate HFD‐Induced Decreases of Glucose Tolerance and Insulin Resistance

**FIGURE 1:**
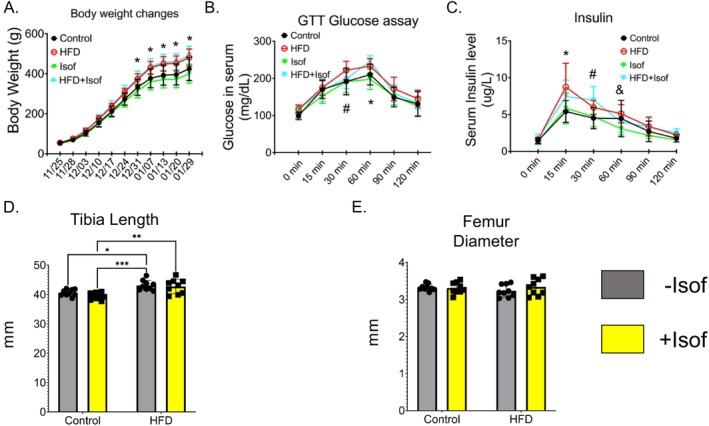
HFD increased body weight, disrupted glucose metabolism and insulin resistance. (A) Body weight gains (growth curves) from four different diet groups. Significant increases in body weight were observed for HFD and HFD + Isof rats compared to Control and Control + Isof rats 4 weeks after start of experiment (indicated by *) (B) Serum glucose concentrations after OGTT for PND61 rats. Animals were fasted overnight, and tail blood was sampled over a 150‐min period following a 2.5 g/kg oral glucose challenge. At 30 min, glucose levels for HFD rats were significantly higher than those for both Control and Control + Isof rats (indicated by #). At 60 min, glucose levels for HFD and HFD + Isof rats were significantly increased compared to both Control and Control + Isof rats (*). (C) Serum insulin concentration after OGTT in PND61. At 15 min, insulin levels for HFD and HFD + Isof rats were significantly increased compared to Control and Control + Isof rats (*). However, at 30 min, a significant difference in insulin concentration was detected only between HFD and Control/Control + Isof rats (#). Finally at 45 min, the difference in insulin concentration between HFD and Control + Isof rats was deemed significant (indicated by &). (D) Rat tibia length (mm) from different dietary groups. Tibias from HFD and HFD + Isof rats were shown to be significantly longer than those from Control and Control + Isof rats. (E) Rat femur diameter (mm) from different dietary groups. No significant differences among dietary groups were detected. All data is presented as mean ± SD (*N* = 9). For two‐way ANOVA followed by Student–Newman–Keuls post hoc analysis for multiple comparisons was performed for bar graphs (E, F) *p ≤ 0.05, **p ≤ 0.01, ***p ≤ 0.001, ****p ≤ 0.0001.

In our current study, male rats were fed diets with low or high fat made with either casein or supplemented with Isof, as described in the methods. After 4 weeks, body weight was significantly increased (*p* < 0.05) for both HFD and HFD + Isof rat dietary groups when compared to Control or Control + Isof rats. Body weights for HFD and HFD + Isof rats were shown to be consistently higher than those for Control and Control + Isof rats for each date (Figure 1A; File S1). Body weight gains in HFD and HFD + Isof rats suggest Isof‐containing diets do not protect against HFD‐induced weight gains. At 30 min, serum glucose levels for HFD rats were significantly higher than for Control or Control + Isof rats. While at 60 min, HFD and HFD + Isof rat serum glucose levels were significantly higher when compared to the other two dietary groups (Figure 1B). At 15 min post glucose challenge, serum insulin levels were found to be significantly increased for both HFD and HFD + Isof rats compared to Control ± Isof rats. At 30 min, insulin levels for HFD rats were significantly higher than for Control and +/− Isof rats. At 45 min, serum insulin levels were significantly increased compared to only Control + Isof rats (Figure 1C). It was found that tibias for HFD and HFD + Isof rats were significantly longer than tibias from Control and Control + Isof rats (Figure 1D). However, no significant differences for rat femur diameter were detected between the dietary groups (Figure 1E).

### 3.2 Isof Prevents HFD‐Induced Impairments of Bone Quantity and Quality

**FIGURE 2:**
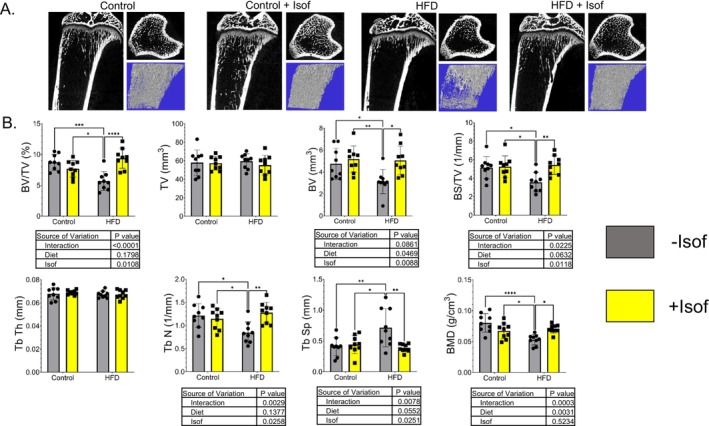
Soy Isoflavones prevented HFD‐induced decreases of bone volume, density, and trabecular number. (A) Representative images of quantitative μCT analysis of tibia trabecular bone in Control, Control + Isof, HFD, and HFD + Isof rats. (B) Bone volume/tissue volume, BV/TV (%); Total tissue volume, TV (mm^3^); Total bone volume, BV (mm^3^); Bone surface/tissue volume, BS/TV (1/mm); Trabecular thickness, Tb. Th (mm); Trabecular number, Tb. N (no./mm); Trabecular spacing, Tb. Sp (mm); Bone mineral density, BMD (g/cm^3^). All data is presented as mean ± SD (*N* = 9). For two‐way ANOVA, followed by Student–Newman–Keuls post hoc analysis for multiple comparisons, *p ≤ 0.05, **p ≤ 0.01, ***p ≤ 0.001, ****p ≤ 0.0001.

The μCT analysis of tibial bones revealed the effects of HFD and Isof dietary supplementation on bone phenotype. As shown by the representative images, trabecular bone was decreased in HFD rat tibia compared to all other rat dietary groups (Figure 2A). From the μCT parameters, BV/TV, BV, BS/TV, Tb N, and BMD were significantly lower in the HFD rats compared to Control, Control + Isof, and HFD + Isof rats; while Tb Sp was significantly higher for HFD rats compared to Control, Control + Isof, and HFD + Isof rats (Figure 2B). TV and Tb Th did not show significant differences between all four rat groups (Figure 2B). Micro‐CT analysis of L3–L5 vertebrae and tibia cortical bone was also performed. However, μCT analysis revealed no significant change in either L3–L5 vertebrae or tibia cortical bone quantity/quality (Figure S1; File S2).

**FIGURE 3:**
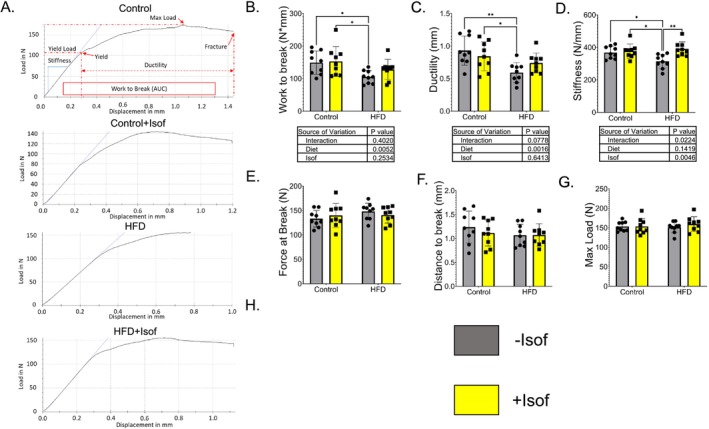
Soy Isoflavones prevented HFD‐induced decrease of bone strength—biomechanical testing of rat femurs using three‐point bending method. (A) Represented load displacement curves from three‐point bending analysis of Control, Control + Isof, HFD, and HFD + Isof rats. (B–G) Three‐point bending analysis parameters from Control, Control + Isof, HFD and HFD + Isof rat femurs. Significant decreases in Work to break (B), Ductility (C) and Stiffness (D) were observed for HFD rats compared to all other dietary groups. Stiffness was brought back to Control and Control + Isof diet levels for HFD + Isof rats. For Force at Break (E), Distance to break (F), Max Load (G) and Yield Load (H) differences between groups were non‐significant. All data is presented as mean ± SD (*N* = 9). For two‐way ANOVA, followed by Student–Newman–Keuls post hoc analysis for multiple comparisons *p ≤ 0.05, **p ≤ 0.01, ***p ≤ 0.001, ****p ≤ 0.0001.

Three‐point‐bending test was performed on femurs from all rat diet groups. A representative load displacement curve from each dietary group is presented in Figure 3A. From the parameters we have analyzed, we found work to break in HFD rat femurs was significantly lower compared to Control and Control + Isof diet rats. The work to break increased for HFD + Isof rats compared to HFD rats, but it did not reach significance (Figure 3B). For ductility, results were similar to work to break (Figure 3C). Femur stiffness of Control, Control + Isof, and HFD + Isof rats was significantly greater than that of HFD rats (Figure 3D). No statistical differences were detected among different rat dietary groups for force at break (Figure 3E), distance to break (Figure 3F), max load (Figure 3G), and yield load (Figure 3H).

**FIGURE 4:**
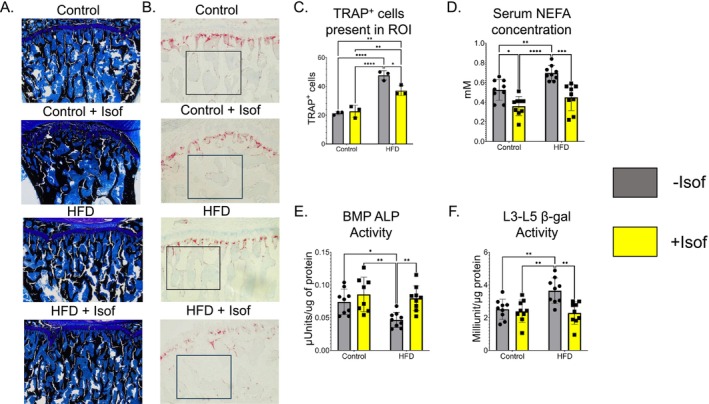
HFD‐induced increase in TRAP+ cells, serum NEFA concentration, as well as ALP and β‐gal activity is improved to near control diet levels by Soy Isoflavones. (A) Representative images of rat tibia bone cavity from each dietary group following Von Kossa staining at 4X magnification. (B) Representative images of rat tibia bone cavity from each dietary group following TRAP staining at 10X magnification. From 10X magnification, region best representative of area under the growth plate (black box) was selected for quantification TRAP^+^ cells present on the slide. (C) Quantification TRAP^+^ cells (in red) present within ROI (*N* = 3). TRAPase^+^ cell number was normalized to ROI area, approximately 893 450 μm^2^. (D) NEFA (non‐esterified fatty acid) concentration in rat serum. (E) ALP (alkaline phosphatase) activity in bone marrow plasma from rat femur (*N* = 9). (F) β‐Gal (senescence‐associated beta‐galactosidase) activity in L3‐L5 vertebrae protein lysate (*N* = 9). All data is presented as mean ± SD. For two‐way ANOVA, followed by Student–Newman–Keuls post hoc analysis for multiple comparisons *p ≤ 0.05, **p ≤ 0.01, ***p ≤ 0.001, ****p ≤ 0.0001.

Based on von Kossa staining, tibia bone mineralization appears similar for all rat dietary groups (Figure 4A). TRAPase staining revealed a significant increase in the number of osteoclastic TRAPase^+^ cells present in tibia bone marrow of HFD rats when compared to the other dietary groups. TRAPase^+^ cell number for HFD + Isof rats was significantly decreased compared to HFD rats but still significantly higher when compared to both Control and Control + Isof rats (Figure 4B,C). Serum NEFA levels were significantly increased for HFD rats compared to Control, Control + Isof, and HFD + Isof rats (Figure 4D). This trend was also observed for serum NEFA levels (Figure 4E). Measurement of the activity of the bone formation marker ALP in bone marrow plasma revealed a significant decrease in ALP activity for HFD rats compared to Control, Control + Isof, and HFD + Isof rats (Figure 4E) [32]. Finally, the activity of senescence‐associated protein β‐Gal [12] was measured in L3‐L5 vertebrae protein lysates, and it was found that β‐Gal activity was significantly higher in HFD fed rats compared to Control, Control + Isof, and HFD + Isof rats (Figure 4F).

### 3.3 Isof Blocks HFD‐Induced Increase of Ezh2 Expression and DNA Methylation in Bone

**FIGURE 5:**
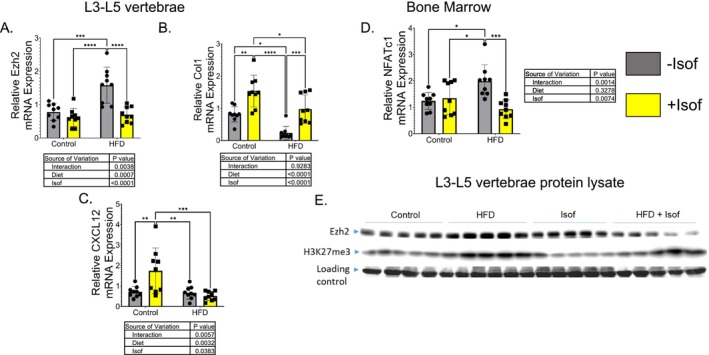
Soy Isoflavones blocked HFD‐induced increase of Ezh2 expression and altered mRNA levels of osteogenic and osteoclastic markers. Total RNA and protein were isolated from L3 to L5 vertebrae after removing surrounding connective tissues. Real‐time PCR shows (A) Ezh2, (B) Col1, and (C) CXCL12 mRNA levels in bone tissue in rats from each dietary group. Total RNA was also isolated from femur bone marrow to better investigate the effects of isoflavone on bone cell gene expression. Real‐time PCR shows (D) NFATc1 mRNA levels in bone marrow from rats from each dietary group. Real‐time PCR data is presented as mean ± SD (*N* = 9). For two‐way ANOVA, followed by Student–Newman–Keuls post hoc analysis for multiple comparisons *p ≤ 0.05, **p ≤ 0.01, ***p ≤ 0.001, ****p ≤ 0.0001. (E) Western blots showing change in Ezh2, H3K27me3, and levels from L3‐L5 vertebrae protein lysate. Five samples, from random pooling, were used for each dietary group.

We have previously reported that maternal HFD upregulates Ezh2 expression, increasing the presence of the H3K27me3 repressive epigenetic mark to change the expression of genes involved in bone tissue remodeling [22, 23]. In L3‐L5 vertebrae, Ezh2 mRNA levels were significantly increased for HFD rats compared to Control, Control + Isof, and HFD + Isof rats (Figure 5A). In addition, L3‐L5 vertebrae mRNA levels for type 1 collagen (Col1) were significantly increased for Control + Isof rats compared to all other rat dietary groups, while for HFD rats, Col1 mRNA levels were significantly decreased compared to Control, Control + Isof, and HFD + Isof rats (Figure 5B). Finally, in L3‐L5 vertebrae, mRNA levels for stromal cell derived factor 1 (CXCL12) were significantly increased for Control + Isof rats compared to all other dietary groups, with no other significant differences detected between groups (Figure 5C). To investigate the effect of HFD and Isof dietary supplementation on changing the expression of genes associated with bone resorption or bone formation at the cellular level, bone marrow, which contains both osteoclast and osteoblast precursors, was isolated from the femur and RNA was extracted. In bone marrow, mRNA levels for the osteoclastogenesis‐associated gene, Nuclear Factor of Activated T cells 1 (NFATc1), were significantly increased for only HFD rats compared to all rat dietary groups (Figure 5C,D). For real‐time PCR, the data were normalized against 18S rRNA. Finally, protein lysate from L3‐L5 vertebrae was used for western blots to demonstrate that HFD rats' Ezh2 and H3K27me3 levels were significantly higher than those of Control and Control + Isof diet rats and that Isof dietary supplementation blocks HFD‐induced increased expression of Ezh2 and H3K27me3 (Figure 5E).

**FIGURE 6:**
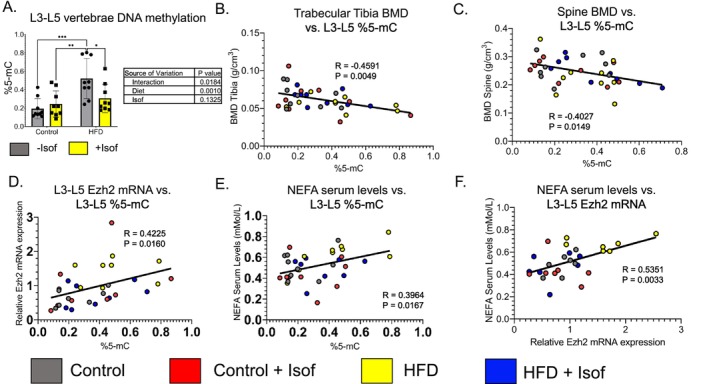
HFD increase in DNA methylation is blocked by Soy Isoflavones and the inverse relationship between BMD and %5‐mC may be due to a positive correlation between serum NEFA and Ezh2 expression. (A) HFD was shown to significantly increase %5‐mC levels in DNA isolated from L3‐L5 vertebrae, while L3‐L5 vertebrae from HFD + Isof fed rats had %5‐mC levels similar to Control and Control + Isof fed rats. For %5‐mC levels, data is presented as mean ± SD (*N* = 9). For two‐way ANOVA, followed by Student–Newman–Keuls post hoc analysis for multiple comparisons *p ≤ 0.05, **p ≤ 0.01, ***p ≤ 0.001, ****p ≤ 0.0001. (B, C) %5‐mC from L3 to L5 vertebrae was shown to have significant moderate negative correlations with both tibia and L3‐L5 vertebrae BMD (*N* = 9). (D, E) %5‐mC levels from L3‐L5 vertebrae were found to have significant moderate positive correlations with both Ezh2 mRNA (*N* = 8) and NEFA serum levels (*N* = 9). (F) NEFA levels in serum were shown to have significant moderate positive correlation with Ezh2 mRNA levels (*N* = 7). For correlations, outliers were removed and statistical analysis was performed using the Correlation function in GraphPad Prism 9.0.

Using a methylflash colorimetric global DNA methylation kit, we also measured the percentage of 5‐methylcytosines (%5‐mC), a marker of DNA methylation, in DNA isolated from L3‐L5 vertebrae from each rat dietary group. HFD rats were shown to have a significantly increased %5‐mC compared to all other dietary groups, with no other significant differences detected (*p* < 0.05) (Figure 6A). To determine if HFD or Isof dietary supplementations' effects on bone may be through altering DNA methylation levels to change the expression of bone resorption/bone formation related genes, the presence of correlations between %5‐mC and BMD, as well as Ezh2 mRNA levels and serum NEFA levels, was determined. Moderate but significant (*R* = −0.6 – −0.4; *p* < 0.05) negative correlations were found between %5‐mC and tibia BMD (*R* = −0.4591) (Figure 6B) and L3‐L5 vertebrae BMD (*R* = −0.4207) (Figure 6C), while moderate but significant (*R* = 0.4–0.6; *p* < 0.05) positive correlations were found between %5‐mC and L3‐L5 vertebrae Ezh2 mRNA levels (*R* = 0.4225) (Figure 6D), %5‐mC and serum NEFA concentration (*R* = 0.3964) (Figure 6E), and L3‐L5 vertebrae Ezh2 mRNA levels and serum NEFA concentration (*R* = 0.5351) (Figure 6F).

## 4 Discussion

### 4.1 Impact of HFD on Bone Metabolism

In this study, we have provided additional evidence showing the negative effects of chronic HFD consumption on bone metabolism in post‐weanling juvenile rat models. Results from our current study support previous observations that disrupted glucose metabolism and insulin resistance observed in rats fed a HFD may be due to elevated NEFA [6]. The effects of Isof dietary supplementation on HFD‐induced body weight gains, glucose tolerance, and insulin resistance were minimal when compared to previous SPI diet studies [6]. Previous studies have shown that isoflavones may not impact glucose metabolism and insulin resistance, which suggests additional factors in the SPI diet outside of isoflavones may explain the discrepancy observed between this study and our previous SPI diet results [6]. The use of alginate—a common stabilizer, solubilizer, and emulsifier in food systems—with SPI results in the formation of gel in the stomach acid that lowers postprandial glucose response by delaying gastric emptying [33]. In addition, soy proteins/peptides may act on different molecular targets associated with glucose response including glucosidase and amylase enzymes [34, 35]. Further research is needed to fully understand additional factors in the SPI diet responsible for regulating glucose metabolism and insulin resistance.

### 4.2 Isof's Effects on Structural and Cellular Bone Parameters

While there may be other factors from the SPI diet involved in glucose metabolism/insulin resistance using μCT, three‐point bending analysis, histological staining, analysis of bone turnover markers, and real‐time PCR, we have found that Isof dietary supplementation can suppress HFD‐induced bone deterioration at both the tissue structure and cellular composition levels. μCT results suggest HFD negatively impacts bone remodeling, as evidenced by decreased BS/TV [36]. Decreased remodeling would decrease the deposition of new trabecular bone in the tibia, leading to decreased Tb N and increased Tb Sp, which in turn would lead to decreased bone volume and density, as seen from decreased BV/TV, BV, and BMD. Results for three‐point bending analysis show a decrease in whole bone strength for HFD rat femurs, as evidenced by decreased work to break, ductility, and stiffness for HFD rats compared to both Control and Control + Isof rats. For HFD + Isof rats, only femur stiffness was significantly increased back to near Control and Control + Isof levels, which suggests that Isof's positive effects on cortical bone may be more diminished when compared to trabecular bone. The overall effect of Isof dietary supplementation on bone mechanical properties in rodents will need to be investigated further in future studies. Although we have presented data showing that tibia length was significantly longer in HFD rats compared with control diet rats, we do not have data to support whether such a change in bone length in rodent models is biologically relevant and if such effects can be observed in humans. Further investigations will be needed to provide a mechanistic explanation for differences in tibia length due to diet.

Based on von Kossa staining, HFD or Isof dietary supplementation did not affect bone mineralization when compared to control rats. However, TRAPase staining and quantification of bone turnover markers ALP and β‐gal reveal a shift in HFD rats toward bone resorption when compared to the other dietary groups. Excess serum NEFA, due to HFD, is likely deposited as adipose tissue, explaining increased body weight for both HFD and HFD + Isof rats. Increased bone marrow adipose tissue would potentially increase inflammation, which would result in increased osteoclastogenesis and bone resorption [37, 38]. Isof supplementation of HFD significantly decreased the number of TRAPase^+^ cells present in bone marrow, though not to the same level as Control or Control + Isof rats. The partial decrease in TRAPase^+^ cells for HFD + Isof rats may in part be due to the adipose tissue suppressing effects of soy isoflavones, but suggests additional osteoclastogenesis promoting effects from HFD other than increased adipose tissue formation [12, 23, 39, 40, 41]. While von Kossa staining shows an increase in the appearance of what are potentially adipose‐tissue (white areas) for HFD rat tibia that could be quantified, it cannot be confirmed that these regions are in fact adipose tissue [42]. As such, additional histomorphometric staining (Oil Red O, H&E, PLIN1 immunohistological staining) will need to be performed to confirm the effects of both HFD and Isof dietary supplementation on bone marrow adiposity [43]. In addition, higher magnification will be used to assess the effects of diets on bone cell morphology.

CXCL12, whose expression has previously been shown to be increased by low concentrations of soy isoflavones, can promote bone formation by promoting recruitment and subsequent maturation of osteoblast precursors in the bone marrow and would help to partially explain the bone anabolic effects of Isof dietary consumption [44, 45]. While HFD has previously been associated with an increase in hematopoietic stem cells (osteoclast precursors), which would explain the increase in bone resorption [46]. The decrease in bone resorption back to control diet levels for HFD + Isof rats may be due to adipose tissues suppressing the effects of isoflavones [12, 39, 40].

Isof may promote collagen production due to its ability to mimic estrogenic effects [47]. Consumed Isof, including genistein or daidzein, is in an inactive glycoside form and must be converted by the gut microbiome to active aglycone forms, which are more bioactive and can be processed further by the gut microbiome to exert their effects. The knowledge of microbiota species responsible for the activation of isoflavones into aglycone forms remains scarce. However, *Adlercreutzia equolifaciens* , *Asaccharobacter celatus* , *Enterorhabdus mucosicola* , *Slackia isoflavoniconvertens* , *Slackia equolifaciens* , and other related bacteria were found to be activators of Isof [48]. Further knowledge on microbiota species responsible for Isof activation and processing is necessary as it may be that only those with certain microbiome compositions may be able to fully process dietary Isof. Processed Isof may interact with estrogen receptors, Erα and Erβ, to regulate bone remodeling. Genistein has previously been shown to have a stronger affinity for Erβ than Erα [49]. Erβ may significantly enhance ALP activity and osteocalcin levels, increasing osteoblast differentiation [50, 51]. Both Erα and Erβ have been shown to suppress adipocyte differentiation in bone tissue [50]. In addition to promoting osteoblast differentiation, Erβ can negatively regulate osteoclast levels and activity by inducing apoptosis and inhibiting bone resorption through pathways that have yet to be fully defined [52].

### 4.3 Isof Epigenetic Activity May Ameliorate HFD Induced Bone Deterioration

While future studies will focus on investigating genes regulated by either HFD or Isof dietary supplementation in rodents, we have presented evidence that Isof supplementation of HFD downregulates Ezh2 expression back to control diet levels, which would suppress levels of H3K27me3, changing gene expression for osteogenesis‐promoting and osteoclast‐suppressing genes, which may explain the suppressed negative effects of HFD on bone [3, 22]. Isof supplementation effects on Ezh2 were only apparent in the presence of HFD; Ezh2 mRNA and protein levels in bone tissue from Control + Isof rats were similar to those in Control rats. Genistein has previously been shown to suppress Ezh2 activity through phosphorylation [53]. However, the mechanisms by which genistein may suppress Ezh2 mRNA and protein expression are currently unknown and will need to be investigated in future studies. Ezh2 has previously been shown to negatively regulate expression of type I collagens; as such, the decrease in *Col1* mRNA levels for HFD rats and subsequent recovery to near Control diet levels for HFD + Isof rats is reasonable [54]. Ezh2 is known to indirectly promote induction of NFATc1 through silencing of *Irf8*, potentially further explaining the increase in *NFATc1* mRNA levels [55]. While Ezh2 levels have been shown to be upregulated with obesity, the specific link between NEFA and Ezh2 expression/activity is currently unknown and will need to be investigated in future studies.

Significant increase for bone tissue DNA methylation in HFD rats compared to Control and Control + Isof rats may be due to the formation of Ezh2/DNA methyltransferase epigenetic gene silencing complex [56]. Subsequent decrease in DNA methylation in bone tissue from HFD Isof rats back to Control +/− Isof levels may be due to suppressing Ezh2 activation. It may also be that HFD induces additional epigenetic changes resulting in gene silencing aside from Ezh2/H3K27me3 activation. Studies focusing on the link between NEFAs and DNA methylation as well as DNA methylation and bone turnover are limited. However, DNA methylation levels were found to be increased in bovine oocytes and embryos following exposure to NEFA [57]. In addition, our group found significant increased DNA methylation in the promoter region of *HoxA10*, a part of a family of transcription factors known to be directly involved in osteoblast differentiation, in fetal calvaria cells from rat HFD fed mothers [58]. For stem cells undergoing osteogenesis, hypomethylation was observed in the promoter regions of multiple osteogenic genes [59]. In addition, DNA methylation inhibitor 5‐Aza‐dC was found to promote osteogenesis of MG‐63 cells [60]. DNA demethylation ability of Isof is well known. It may be that Isof dietary supplementation of HFD prevents bone deterioration by reversing HFD induced DNA hypermethylation, leading to increased expression of osteogenesis and osteoclast suppressing genes [61].

### 4.4 Study Limitations and Future Directions

It should be noted that there are several limitations to this study. First, the effect of Isof dietary supplementation on bone was investigated in only male rats. While the sex‐specific differences in obesity‐induced pre‐pubertal bone loss are not fully understood, we and others have found that the negative effects of HFD on bone in post‐weanling juvenile rodents are more pronounced in males than in females [62, 63]. Early life nutritional factors may protect against bone deterioration later in adulthood (dietary‐induced, sex steroid deficiency, etc.) by maximizing early life bone formation. Previous research from our group has found that in female rats, SPI diet can ameliorate ovariectomy (OVX)‐induced bone loss through a different mechanism than 17β‐Estradiol injection [10, 11]. Future efforts into ameliorating HFD‐induced effects on bone in post‐weanling juvenile female rodents may provide strategies for minimizing late‐life sex steroid deficiency‐induced bone loss in women and may highlight novel sex differences in early life bone development between males and females.

Second, the potential “feminizing” effects (decreased testosterone, increased estrogen, development of female sex characteristics) of Isof dietary supplementation on male rats were not investigated. While isoflavones can bind to estrogen receptors, their effects are much weaker than estrogen and numerous studies have found that isoflavones have no significant effect on estrogen or testosterone levels. However, it may be that excessive, long‐term consumption of soy isoflavones has “feminizing” effects as some case reports have noted hypogonadism and the development of female sex characteristics in older men (50–60 years of age) [64]. Due to this, the potential “feminizing” side effects of Isof dietary supplementation on male rodents will need to be investigated further in future studies.

Finally, the link between DNA methylation changes and bone protective effects of Isof supplementation of HFD is purely speculative. The investigation into bone tissue DNA methylation changes among the diets focused only on global changes in %5‐mC and not changes in the promoter or gene body regions of specific osteogenic or osteoclastic genes. The correlations measured in this study (BMD and %5‐mC, Ezh2 mRNA and %5‐mC, serum NEFA levels and %5‐mC, Ezh2 mRNA and serum NEFA levels) while significant (*p* < 0.05), were only moderate suggesting that Isof epigenetic reprogramming via suppression of Ezh2 may alleviate some of the damage to bone from HFD but that there may be changes to bone metabolism from HFD that Isof dietary supplementation cannot suppress. Future studies will need to investigate specific mechanisms surrounding Isof bone protective effects. Specific experiments (bisulfite sequencing, methylation specific real‐time PCR) will be performed to determine gene regulated by Isof induced DNA hypomethylation. In addition, RNA‐seq, microarray analysis and protein array analysis experiments will need to be performed to identify bone formation/resorption pathways regulated in rodents following HFD or Isof dietary supplementation. ChIP‐seq experiments performed on bone tissue or bone cells (osteoblasts or osteoclasts) might also provide additional details regarding Isof induced regulation of Ezh2/H3K27me3 gene silencing.

### 4.5 Conclusions

Diets rich in saturated fats have become a staple globally, greatly contributing to obesity in the US [3, 4]. A previous human study has demonstrated an inverse association between fat mass and bone mineral content (BMC) [65]. However, studying the relationship between HFD consumption and bone health in humans represents a significant challenge as it would require many years of follow‐up, leaving room for multiple factors to potentially contribute to bone loss and the potential development of osteoporosis. We have provided data indicating that dietary supplementation of HFD with soy Isof protects against bone loss, potentially through suppression of HFD‐induced Ezh2 expression and DNA hypermethylation in bone. While stopping chronic consumption of HFD is optimal for preventing further metabolic deterioration, it may not be enough to reverse the negative and long‐term metabolic outcomes associated with chronic HFD consumption. As such, Isof dietary supplementation may be an effective approach for protecting against the long‐term metabolic consequences of chronic HFD consumption on bone. Such a strategy may be necessary for young populations that primarily consume ‘Western’ style diets, high in fat, to prevent bone deterioration and the development of bone resorption disorders in adulthood [65, 66, 67].

## Author Contributions

J.‐R.C. designed research; P.C.C. and J.‐R.C. analyzed data and wrote the paper; B.B., O.P.L., J.F.C., and M.L.B. performed research; J.F.C. analyzed data.

## Conflicts of Interest

The authors declare no conflicts of interest.

## Supporting information


**Table S1:** Real‐time reverse‐transcription polymerase chain reaction (RT‐PCR) primer sequences.

**Figure S1:** Micro‐CT analysis of L3‐L5 vertebrae shows diet did not significantly impact bone quantity or quality. (A) Representative images of quantitative micro‐CT analysis of L3‐L5 vertebrae from four different dietary groups. (B) Bone volume/tissue volume, BV/TV (%); (C) Total tissue volume, TV (mm^3^); (D) Total bone volume, BV (mm^3^); (E) Bone surface/tissue volume, BS/TV (1/mm); (F) Trabecular thickness, Tb. Th (mm); (G) Trabecular number, Tb. N (no./mm); (H) Trabecular spacing, Tb. Sp (mm); (I) Bone mineral density, BMD (g/cm^3^). Data are expressed as mean ± SD (*N* = 9). For two‐way ANOVA, followed by Student–Newman–Keuls post hoc analysis for multiple comparisons *p ≤ 0.05, **p ≤ 0.01, ***p ≤ 0.001, ****p ≤ 0.0001.

## Data Availability

The data that support the findings of this study are available in the methods and/or supplemental material of this article.
