# Supplementary material for: Soy Isoflavones Prevent Bone Quality Loss Induced by High‐Fat Diet in Rats Through Epigenetic Modifications
Source: FASEB J. 2025 Oct 22;39(20):e71158. doi: 10.1096/fj.202500767RRR (PMC12541691; doi:10.1096/fj.202500767RRR)
Supplement: Supplementary file 1 — Table S1: Real‐time reverse‐transcription polymerase chain reaction (RT‐PCR) primer sequences. [file FSB2-39-e71158-s002.docx]

**Supplemental Table 1**. Real-Time Reverse-Transcription Polymerase Chain Reaction (RT-PCR) Primer Sequences.

| Gene | Forward Primer | Reverse Primer |
| --- | --- | --- |
| Ezh2 | CTTTTGTGCCATTGCTAGGCTAA | CCTCAGTGGGAACAGGTGCTA |
| Col1 | TCTCCATGGCCTCTGCAACAAA | TGCACATGTGTGGCCGATGTTT |
| CXCL12 | GAAGTGATCCCTGAAGCTGTGC | GAGGTGAGAAGCGGAAGTCAGA |
| NFATc1 | ACCAAAGTCCTGGAGATCCCA | CCCTTTCCTCAGCTCGATATCA |
| 18S | CCTGTAATTGGAATGAGTCCACTTT | ATACGCTATTGGAGCTGGAATTACC |
