# Supplementary material for: Soy Isoflavones Prevent Bone Quality Loss Induced by High‐Fat Diet in Rats Through Epigenetic Modifications
Source: FASEB J. 2025 Oct 22;39(20):e71158. doi: 10.1096/fj.202500767RRR (PMC12541691; doi:10.1096/fj.202500767RRR)
Supplement: Supplementary file 2 — Figure S1: Micro‐CT analysis of L3‐L5 vertebrae shows diet did not significantly impact bone quantity or quality. (A) Representative images of quantitative micro‐CT analysis of L3‐L5 vertebrae from four different dietary groups. (B) Bone volume/tissue volume, BV/TV (%); (C) Total tissue volume, TV (mm3); (D) Total bone volume, BV (mm3); (E) Bone surface/tissue volume, BS/TV (1/mm); (F) Trabecular thickness, Tb. Th (mm); (G) Trabecular number, Tb. N (no./mm); (H) Trabecular spacing, Tb. Sp (mm); (I) Bone mineral density, BMD (g/cm3). Data are expressed as mean ± SD (N = 9). For two‐way ANOVA, followed by Student–Newman–Keuls post hoc analysis for multiple comparisons *p ≤ 0.05, **p ≤ 0.01, ***p ≤ 0.001, ****p ≤ 0.0001. [file FSB2-39-e71158-s001.pptx]

## Slide 1
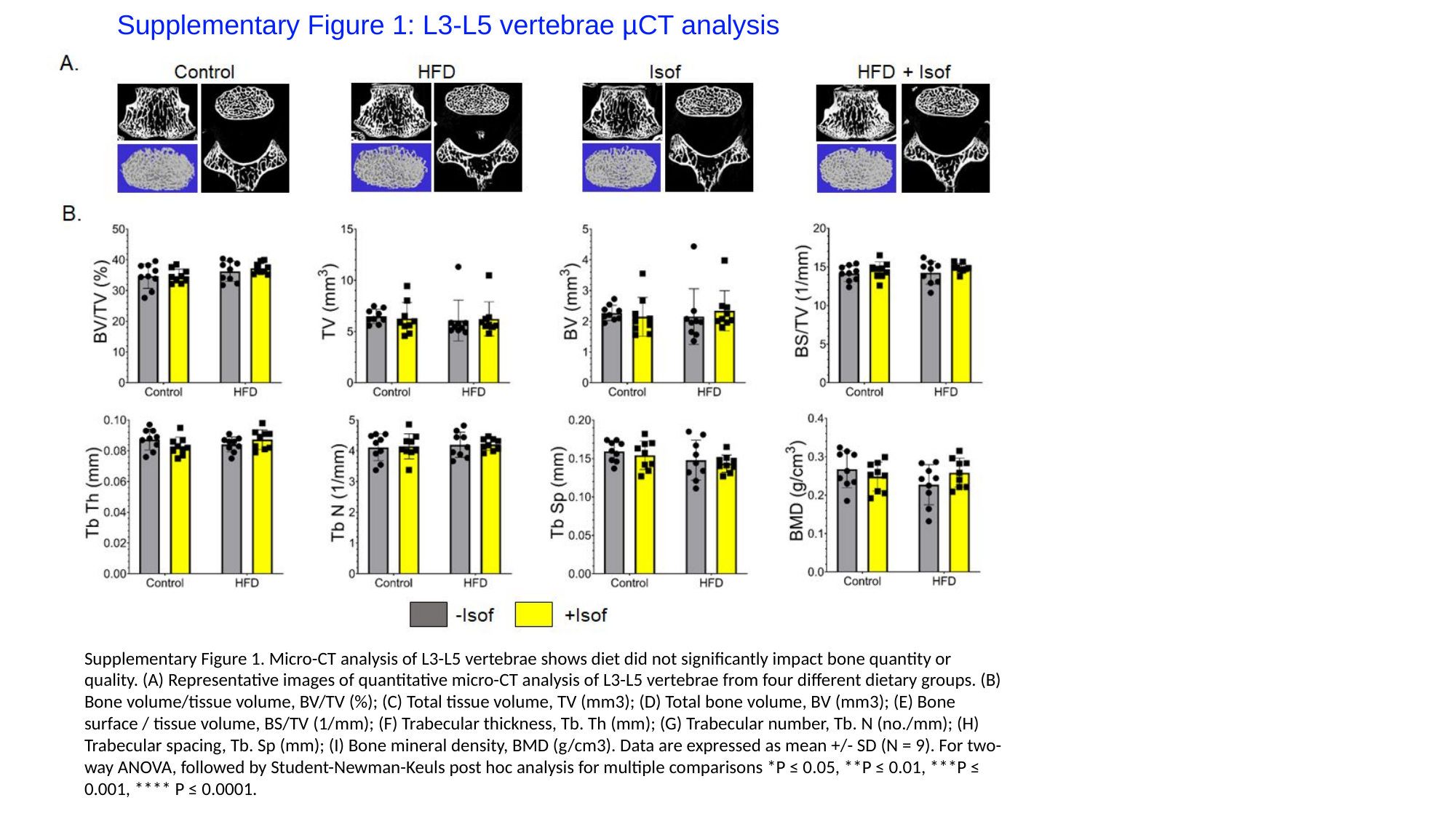

Supplementary Figure 1: L3-L5 vertebrae µCT analysis
Supplementary Figure 1. Micro-CT analysis of L3-L5 vertebrae shows diet did not significantly impact bone quantity or quality. (A) Representative images of quantitative micro-CT analysis of L3-L5 vertebrae from four different dietary groups. (B) Bone volume/tissue volume, BV/TV (%); (C) Total tissue volume, TV (mm3); (D) Total bone volume, BV (mm3); (E) Bone surface / tissue volume, BS/TV (1/mm); (F) Trabecular thickness, Tb. Th (mm); (G) Trabecular number, Tb. N (no./mm); (H) Trabecular spacing, Tb. Sp (mm); (I) Bone mineral density, BMD (g/cm3). Data are expressed as mean +/- SD (N = 9). For two-way ANOVA, followed by Student-Newman-Keuls post hoc analysis for multiple comparisons *P ≤ 0.05, **P ≤ 0.01, ***P ≤ 0.001, **** P ≤ 0.0001.
